# Abstract of the Proceedings of the Buffalo Medical Association

**Published:** 1862-04

**Authors:** J. F. Miner

**Affiliations:** Secretary


ART. II—*Abstract of the Proceedings of the Buffalo Medical Association.*

TUESDAY EVENING, March 4, 1862.

The Association met at the usual hour.

Minutes of the last meeting were read and approved.

*Dr. Eastman* was called on the evening of October 10, 1861, to attend Mrs. J——, in her fifth confinement. In all her previous confinements, version had been performed, from malpresentation of the child. He attended her in her fourth and fifth labor, and when summoned to her bed-side on the occasion of her present illness, visited her with the apprehension of again being obliged to turn the child. She was taken in labor at 1 P. M.; there was not much pain till 9 P. M., when he found her in severe labor, the pains occurring with slight intermission. The os uteri was fully dilated; it was impossible to diagnose the presentation, as the membranes had not ruptured. Her suffering being severe, after an hour's interval, he passed his fingers into the vagina, nearly filling the passage, and ruptured the membranes. The waters flowed gradually away, the hand preventing their speedy escape. Part of the head of the child could be felt, when another pain succeeding in a few minutes caused a small part of the funis to descend into the vagina, and before it could be replaced a large fold came down.—Having read Dr. T. Gaillard Thomas' lecture on Prolapse of the Funis, in the September number of the *American Medical Monthly*, he at once determined to follow the "postural treatment" adopted by him. Holding the cord in the vagina, he placed his patient on her knees, using a pillow under each knee to elevate the pelvis; her chest and shoulders resting on the farther side of the bed. He introduced his hand, and returned the funis into the uterine cavity, carrying it past the head of the child and the pelvic brim, beyond the promonitory of the sacrum. The cord did not again come down, as the succeeding pains caused the head to descend and occupy the os uteri. The patient remained upon her knees during a few pains, when she lay down, and the labor proceeded in the usual manner, being completed at 1 A. M., of the 11th October.

The child was immediately placed in warm water, and its chest sprinkled with cold water, and in a few minutes respiration was fully established.

*October* 14,—Patient was dismissed, both mother and child requiring no farther attendance.

*Dr. Eastman* remarked that the case was reported as confirmatory of the practice of Dr. T. Gaillard Thomas, who had recently introduced and recommended what he denominated the "Postural Treatment" in cases of prolapse of the funis. Dr. G. N. Burwell, of Buffalo, had also reported a case in which he adopted successfully, the same plan of treatment. Several years since he had treated a similar case, trying to return the cord with the hand but did not succeed; sent for forceps, and delivered the child, which was dead.

*Dr. Gould* had met similar cases; forceps were applied but the children were lost.

*Dr. Gay* had observed cases where the cord was twisted around the neck; thought it a common occurrence.

*Dr. Eastman* had met one such where the cord was three times around the neck, and the secundines were discharged with the child.

*Dr. Gould* spoke of a case of twins where, with the second child was discharged the placenta of the first, the cord wound closely around the leg; the children were both still-born.

*Dr. Gay* spoke of the use of, solution per sulphate of iron, in menorrhagia; had long been in the habit of using an acid solution of sulphate of iron, while the use of the solution of per sulphate had been suggested by Dr. White. Had recently treated an anemic debilitated patient, who menstruated every two weeks; ordinary remedies of no use in controlling the disease; gave 12 drops solution per sul ferri every 4 hours, when patient commenced to improve; is now better than for many years; is perfectly regular. He is testing its value also in leucorrhoea.

*Dr. Gould* has found quinine and elixir vitriol the best remedy.

℞ quinine ʒi.

Elix. vitriol ℥i.

M. 15 drops 3 times daily.

*Dr. Eastman* had recently prescribed, per sul ferri in two cases of albuminuria; one case succeeding scarlet fever, the other puerperal disease. It produced nausea and he was obliged to discontinue it.

*Dr. Miner* called the attention of the Association to the *Treatment of ingrowing toe-nail, by Per Chloride of Iron.* The suggestion was from M. Wahn, physician at the Military Hospital, at Nice, and is published in the *Gazette des Hospitande.* Since this publication he had had, several opportunities of testing its value. A few grains introduced by the side of the nail between the free edge and the ulcer, was the most pleasant and efficient treatment he had ever seen adopted. Had used it thus far in the milder cases only, and with the greatest satisfaction; thought possibly it was better adapted to the early stages of the complaint or to the milder degrees of irritation, inflammation or ulceration. The importance of this condition of the nail was referred to, and the great superiority of the tanning or mummifying process, as it had been called, contrasted with the slicing, cauterising, extracting, or amputating plan so generally adopted in severe cases. One application was generally sufficient, when, in a few days the condensed tissue might be removed, or if allowed to remain was productive of no injury; it would eventually separate leaving a healthy surface.

His object in mentioning these facts was to add the results of his observation to the Journal reports of its value; while doing this, he desired to say that, it was far from his custom to test new remedies or modes of treatment but that he adhered to the old and beaten path until a more feasible plan was well demonstrated to exist. In this case, nothing could be urged as objectionable in first adopting the milder plans of procedure; amputation of the toe or extraction of the nail, might be performed after fair trial had been made with the per chloride ferri.

*Dr. Miner* desired also to report in brief, a case of disease of the knee joint, since the qustion of opening into joints had recently been raised in the Society, on the occasion of his reporting a case visited in Fort Erie, C. W.

F. W., aged 12 years, admitted to the Surgical ward, Buffalo General Hospital, January 3d, 1862. Had received a fall three years since, and had soon after commenced to complain of pain in the knee; it had been swollen considerably for the last two years and the leg flexed upon the thigh. He had made no use of it in walking, using constantly a crutch; he had been treated by two of the oldest and most experienced physicians in the city.

On admission the boy was pale, thin, and anemic in appearance; the pain complained of, as constant and severe; the appetite and general health very poor.

The knee was found greatly swollen and giving the distinct sense of fluctuation; the integument was not much redened and tenderness on pressure was very uniform over the whole surface

With the consent of his colleagues, Drs. Eastman and Lothrop, an exploring trocar and canula was introduced, with the expectation of an escape of serum from the cavity of the knee joint, and of thus lessening the pain and inflammation. The canula being quite small no fluid was obtained. Without further hesitation, an opening was made with scalpel into the cavity of knee joint, when there escaped considerable quantity of pus.

In one week the swelling and pain was greatly lessened, still there remained a diminished sense of fluctuation and with scalpel the spongy, distended structures, were again opened; the discharge of pus was thus rendered for a few days, more free and abundant.

In a short time the leg was dressed upon splint, with extending rod and screw and the flexed joint was straightened without pain or inconvenience.—The pain has almost wholly abated and the boy is around the ward with appearance of finally regaining the partial or complete use of the leg, though the permanency of the improvement must be, in all cases of scrofulous disease, exceedingly uncertain.

This case had been given, not so much on account of any wonderful or unusual result of treatment, or of any striking peculiarity in the case itself, but rather because it is a common one, and similar ones liable to be presented very frequently for advice and treatment, and also for the purpose of saying a word upon the point of opening into joints. This whole subject had been brought before the profession more or less fully, for a few years past, and no doubt there had been some change of views in relation to the danger of wounds penetrating the larger joints, or of openings made by the surgeon in cases of disease. We had said in the former meetings of the Society without any dissenting voice, that where pus had accumulated there could be no differences of opinion; all would agree that it was proper and desirable to make opening and allow it to escape; yet possibly even in this, the views and practice of surgeons would be found not wholly uniform.

Most standard authors speak of the great dangers of such wounds, and of punctures admitting air into large joints; and we are cautioned to make incisions in manner not to allow such accident. Puncture to allow the escape of serum when effused in such situations is hardly considered, and when proposed we are told, air must not be admitted. This fear of air being admitted to joints has been almost universal, and though some surgeons have had less fear of it, than others, still not until the investigations of Dr. Cooper, of San Francisco, and the published reports of his cases had been given to the profession, was there found much difference in opinion.

In hydrarthrosis, Dr. Richard Barwell, recommends in some cases the injection of dilute tincture of iodine, "that it is so seldom followed by too violent inflammation, that it may be used without fear;" yet in about the next sentence after describing the process of injecting, he says:

"It is essential to observe that no *air enter the cavity*, and for this purpose it is necessary that the syringe have a well fitting piston, and be accurately full of the liquid." This is a representative sentiment and speaks the prevailing views of the profession. Dr. Cooper has expressed opposite views and some of the most experienced surgeons of our country have more or less fully endorsed his opinions and adopted the practice thus suggested. *Dr. M.*, did not desire to express any opinion but simply to give the case he had recently observed, and to say that many others were fresh in his recollection, which if recited, would in his opinion, tend to show, that we have less by far to fear in openings made into the cavity of joints, than we have generally been led to suppose.

*Dr. Eastman* reported the case of a ship carpenter, who about three weeks since, struck his broad axe through the patella and into the joint nearly in the median line. The wound was temporarily dressed and he was admitted to the Hospital of the Sisters of Charity about two hours after the accident. Prof. Moore was in the hospital at the time, and gave clinical lecture upon the dangers of wounds into the joints; stated that "they were usually fatal," but finally concluded that the best treatment would be to close the wound with sutures and adhesive plaster, which was done, leaving an open place above and below, for the escape of any accummulation. It was closed without washing away the blood, just as it was found on admission and placed upon a splint and cold water dressings applied; low diet was directed, and anodynes to allay pain. The result is, that it has mostly closed by first intention, only a very little purulent matter having discharged from the lower opening; now, three weeks since the accident, there is every indication of a pleasant recovery.

*Dr. Eastman* remarked that this case was very interesting in connection with the remarks of Dr. Miner, illustrating the various points which had been under discussion by one case directly applicable. These subjects are now doubtless better understood since the report of Dr. Cooper, and the observations of others have been published. *Dr. E.*, has always been in the habit of making openings whenever and wherever there was purulent or other accummulation which seemed to require it; and the patients have been relieved, and generally done well.

*Dr. Miner* gave the following history of a case of imperforate anus: Three weeks since was invited to visit a child on High street, five days old; upon examination it was found that the bowel communicated with the urethra so that after urinating, a very slight discharge of fæcal matter took place from the penis, enough to make it certain of connection with the intestine. The site of the anus was smooth and almost unmarked; crying and straining produced no distinct impulse.

Assisted by Dr. Lothrop, a careful dissection was made in direction of the rectum, passing through muscular tissue one and one-half or two inches in extent and reaching the bowel, which terminated either in a culdesac, or was in considerable degree distended by accummulation of fæcal matter. Very free evacuation of bowels took place. The opening was separated fully with a sponge tent, and no further dressings applied.

The child three weeks from the time of operation seems thriving and quite healthy; the dejections from bowels are frequent and abundant and every function seems quite healthily performed. Nothing more of fæcal matter passes the urethra. In every other respect the child is natural, except the left hand, upon which is no thumb; not the slightest "attempt towards a thumb."

Voted to refer the renting of rooms to the Officers of the Association. with power to act.

Voted to adjourn, to Tuesday evening, April 1st,—annual meeting.

J. F. MINER, Secretary.

